# Differentiating Inbred Mouse Strains from Each Other and Those with Single Gene Mutations Using Hair Proteomics

**DOI:** 10.1371/journal.pone.0051956

**Published:** 2012-12-14

**Authors:** Robert H. Rice, Katie M. Bradshaw, Blythe P. Durbin-Johnson, David M. Rocke, Richard A. Eigenheer, Brett S. Phinney, John P. Sundberg

**Affiliations:** 1 Department of Environmental Toxicology and Forensic Science Graduate Program, University of California Davis, Davis, California, United States of America; 2 Department of Public Health Sciences, University of California Davis, Davis, California, United States of America; 3 Proteomics Core, University of California Davis, Davis, California, United States of America; 4 Department of Research, The Jackson Laboratory, Bar Harbor, Maine, United States of America; University of Westminster, United Kingdom

## Abstract

Mutant laboratory mice with distinctive hair phenotypes are useful for identifying genes responsible for hair diseases. The work presented here demonstrates that shotgun proteomic profiling can distinguish hair shafts from different inbred mouse strains. For this purpose, analyzing the total hair shaft provided better discrimination than analyzing the isolated solubilized and particulate (cross-linked) fractions. Over 100 proteins exhibited significant differences among the 11 strains and 5 mutant stocks across the wide spectrum of strains surveyed. Effects on the profile of single gene mutations causing hair shaft defects were profound. Since the hair shaft provides a discrete sampling of the species proteome, with constituents serving important functions in epidermal appendages and throughout the body, this work provides a foundation for non-invasive diagnosis of genetic diseases of hair and perhaps other tissues.

## Introduction

Inbred laboratory mice are the premier animal model system for studying basic biology and human diseases. Inbred strains resemble multiple discrete samplings of the human population, which make them ideal for finely dissecting genetic differences. Based on their derivation history, haplotypes, and single nucleotide polymorphisms, inbred mouse strains have been divided into 7 groups [1]. Present work investigates whether strains from these groups can be distinguished by their hair shaft proteomes. To this end, such analysis can now exploit previously intractable hair protein components as well as those that are isolable, primarily keratins and keratin associated proteins.

Transglutaminase cross-linked protein in the hair shaft has not been amenable to analysis until the advent of mass spectrometry-based proteomics. Over 300 proteins were identified in this fraction of human hair after tryptic digestion and column chromatographic separation [2], and more recent studies are adding to the inventory [3]. Using a more streamlined shotgun approach, proteomic analysis of the hair shaft permitted distinguishing among three related mouse strains and pointed to a lack of trichohyalin expression as being responsible for defective arrangement of medulla cells in AKR mouse hair [4]. This approach may provide criteria for diagnosis of genetically based hair diseases, including those in humans.

Humans have a variety of hair shaft disorders [5]–[7]. Finding mutant laboratory mice with distinctive phenotypes is useful for identifying the genes responsible for such diseases [8] and may permit testing hypotheses and ultimately therapeutic approaches. As a foundation for future effort, the present study indicates noninvasive shotgun proteomics can reveal hair shaft effects resulting from a single gene mutation. Nevertheless, analysis of the hair shaft presents technical challenges, primarily arising from the high content of closely related insoluble keratins. Thus, adjusting the peptide spectra for protein overlap followed by median normalization and then statistical testing provided an effective route for the present work.

## Materials and Methods

### Mice

Pelage hair from 16 mouse strains and mutant stocks were sampled by clipping at The Jackson Laboratory (Bar Harbor, ME; http://jaxmice.jax.org/). The strains (Tables 1 and 2) were AKR/J-*Soat1^ald^/Soat1^ald^* (strain abbreviation: AK; JR#648), BALB/cByJ (CBy; JR#1026), C3H/HeJ (C3; JR#659), C57BL/6J (B6; JR#664), CAST/EiJ (CAST; JR#928), DBA/1J (D1; JR#670), LP/J (LP; JR#676), MRL/MpJ (MRL; JR#486), MRL/MpJ-*Fas^lpr^*/*Fas^lpr^, Foxq1^sa-J^/Foxq1^sa-J^* (MRL-FX; JR#3896), NOD/ShiLtJ (NOD; JR#1976), NZW/LacJ (NZW; JR#1058), RF/J (RF; JR#682), SB/LeJ-*Lyst^bg^*/*Lyst^bg^, Foxq1^sa^/Foxq1^sa^* (SB; JR#269), STOCK-*a/a,ma/ma, Flg^ft^/Flg^ft^*/J (MAFT; JR#281), STOCK- *Sgk3^fz-ica^*/*Sgk3^fz-ica^*/McirJ (FZ; JR#6135), and WSB/EiJ (WSB; JR#1145) (strains abbreviations obtained from: http://www.informatics.jax.org/external/festing/search_form.cgi; http://jaxmice.jax.org/).

**Table 1:** Mouse Strains with Total Hair Analyzed.*

Abbreviation	Strain
C3	C3H/HeJ
B6	C57BL/6J
CAS	CAST/EiJ
D1	DBA/1J
MRL	MRL/MpJ
MRL-FX	MRL/MpJ-*Fas^lpr^/Fas^lpr^, Foxq1^sa-J^/Foxq1^sa-J^*
NOD	NOD/ShiLt
NZW	NZW/LacJ
SB	SB/LeJ-Lyst^bg^, Lyst^bg^, Foxq1^sa^/Foxq1^sa^
MAFT	STOCK-*a/a, ma/ma, Flg^ft^/Flg^ft^*
WSB	WSB/EiJ

* Given are the mouse strains (4 mice each) and their abbreviations.

**Table 2:** Mouse Strains with Hair Fractions Analyzed.*

Abbreviation	# Analyzed	Strain
AK	6	AKR/J
CBy	6	BALB/cByJ
C3	5	C3H/HeJ
B6	5	C57BL/6J
LP	6	LP/J
MRL-FX	6	MRL/MpJ-*Fas^lpr^/Fas^lpr^, Foxq1^sa-J^/Foxq1^sa-J^*
RF	4	RF/J
SB	6	SB/LeJ- Lyst^bg^, Lyst^bg^, Foxq1^sa^/Foxq1^sa^
MAFT	6S	STOCK-*a/a, ma/ma, Flg^ft^/Flg^ft^*
FZ	2P, 4S	STOCK-*Sgk3^fz-ica^*/*Sgk3^fz-ica^*/McirJ

* Given are the strains sampled as detergent-solubilized (S) and insoluble (P) hair fractions, the abbreviations of the strains and the numbers of individual mice sampled.

### Ethics Statement

Mice were maintained in a temperature-, humidity-, and light cycle- (12:12) controlled vivarium under specific pathogen-free conditions (http://jaxmice.jax.org/genetichealth/index.html). Mice were housed in double-pen polycarbonate cages (330 cm^2^ floor area) at a maximum capacity of four mice per pen. Mice were allowed free access to autoclaved food (NIH 31, 6% fat; LabDiet 5K52, Purina Mills, St. Louis, MO) and acidified water (pH 2.8–3.2). All work was done using protocols approved for this study by The Jackson Laboratory’s Animal Care and Use Committee.

### Digest Preparation

For analysis of total hair, samples (5 mg) were rinsed twice in 2% SDS to remove dust and debris, if any, incubated overnight at 37°C in 0.4 ml of 2% SDS/0.1 M NaHPO_4_/25 mM dithioerythritol and then alkylated for 1 hr with iodoacetamide at room temperature with magnetic stirring. The solubilized and insoluble proteins were recovered together by precipitation with 1 ml of ethanol. Each fraction was rinsed twice with 67% ethanol, once with 0.1 M ammonium bicarbonate and digested at room temperature with reductively methylated bovine trypsin [9], 1% by weight, in fresh 0.1 M ammonium bicarbonate-10% acetonitrile. Trypsin was added at daily intervals for a total of 3 days, at which time the digest was clarified and submitted to mass spectrometric analysis. To determine the effect of cysteine S-aminoethylation, the total hair was treated as above except that reduction was conducted with 25 mM dithioerythritol in 0.4 ml of Tris base for 4 hr at room temperature, and the protein was alkyated by subsequent addition of 6 mg of 2-bromoethylamine with stirring for 6 hr at room temperature [10]. For analysis of insoluble and soluble fractions, 18–22 mg of hair were rinsed in 2% SDS as above and extracted overnight at 70°C in 4 ml of SDS-phosphate-dithioerythritol extraction buffer. Solubilized proteins were separated from particulate matter by centrifugation (5 min at 5,000xg). Insoluble material, amounting to ≈18% of the total protein, was extracted a total of 5 times this way and pulverized by magnetic stirring bars for 1–2 hr during each extraction. Aliquots of the particulate material and solubilized protein fractions were incubated with fresh dithioerythritol, alkylated with iodoacetamide, and digested as above.

### Mass Spectrometry

Salts and polypeptides resistant to elution from C_18_ reversed phase material were depleted from samples by solid phase extraction with Aspire RP30 C_18_ desalting tips (Thermo). The tips were rinsed exhaustively with 60% acetonitrile and then 0.1% trifluoroacetic acid. The sample digests were then loaded and washed with 0.1% trifluoroacetic before elution with 60% acetonitrile. The samples (adjusted to approximately equal peptide amounts by A^280^) were then directly loaded onto an Agilent ZORBAX 300SB C_18_ reverse-phase trap cartridge which, after loading, was switched in-line with a Michrom Magic C_18_ AQ 200 μm × 150 mm nano-LC column connected to a Thermo-Finnigan LTQ ion trap mass spectrometer through a Michrom Advance Plug and Play nanospray source and CTC Pal autosampler. The nano-LC column was used with a binary solvent gradient; buffer A was composed of 0.1% formic acid and buffer B composed of 100% acetonitrile. The 120 min gradient consisted of the steps 2–35% buffer B in 85 min, 35–80% buffer B in 23 min, hold for 1 min, 80–2% buffer B in 1 min, then hold for 10 min, at a flow rate of 2 μl/min, for the maximum separation of tryptic peptides. An MS survey scan was obtained for the m/z range 375–1400, and MS/MS spectra were acquired from the 10 most intense ions in the MS scan by subjecting them to automated low energy CID. An isolation mass window of 2 Da was used for the precursor ion selection, and normalized collision energy of 35% was used for the fragmentation. A two minute duration was used for the dynamic exclusion.

### Protein Identification

Tandem mass spectra were extracted with Xcalibur version 2.0.7. All MS/MS samples were analyzed using X! Tandem (The GPM, thegpm.org; version TORNADO (2010.01.01.4)). X! Tandem was set up to search all “mouse” proteins in the uniprot 2011 protein database, 110 proteins from the cRAP database of adventitious proteins (http://www.thegpm.org/crap/) plus an equal number of reverse sequences (299,914 entries) for total hair samples or IPI 2010 mouse databases for fractionated hair samples (56,957 entries) assuming the digestion enzyme was trypsin. X! Tandem was searched with a fragment ion mass tolerance of 0.40 Da and a parent ion tolerance of 1.8 Da. Iodoacetamide derivative of cysteine was specified in X! Tandem as a fixed modification except in S-aminoethylated samples. Deamidation of asparagine and glutamine, oxidation of methionine and tryptophan, sulfone of methionine, tryptophan oxidation to formylkynurenin of tryptophan and acetylation of the N-terminus were specified in X! Tandem as variable modifications. Scaffold (Scaffold version 3.5.1, Proteome Software Inc., Portland, OR) was used to validate MS/MS based peptide and protein identifications for the fractionated hair samples. Peptide identifications were accepted if they could be established at greater than 80% probability as specified by the Peptide Prophet algorithm (false discovery rate 0.1%) [11]. Protein identifications were accepted if they could be established with at least 95% probability and contained at least 2 identified peptides (false discovery rate 3.3%). Protein probabilities were assigned by the Protein Prophet algorithm [12]. Proteins that contained similar peptides and could not be differentiated based on MS/MS analysis alone were grouped to satisfy the principles of parsimony. Numbers of assigned spectra were tabulated. Assigned spectra for the total (but not fractionated) hair samples were adjusted for shared peptides [13] using a locally developed script [14]. Of the 371 proteins identified by the Scaffold software, the most prevalent 187 are given in Table S1.

### Statistical Methods

For total hair samples, the spectral counts (adjusted for shared peptides) were subjected to median normalization in which the adjusted spectral counts were additively adjusted so that the median spectral count across peptides is the same for each mouse. This operation adjusts for variations in sample amount. Differences in spectra from hair proteomes were then analysed as previously described [4] using over-dispersed Poisson regression (glm function in R), a one-way ANOVA (analysis of variance), but with Poisson regression instead of ordinary least squares. Proteins identified in at least 75% of the samples were analysed (57% of those given in Table S1). A protein was considered to be statistically distinct among strains if the P-value from the comparisons was ≤0.05. For proteins for which the global test of a difference among strains was significant, the Tukey HSD approach was used to identify significant pairwise differences (Tables S2, S5 and S6). The data and the code in R to perform the analysis are available from Dr. Durbin-Johnson. Hierarchical clustering of the mouse strains was performed using the hclust function in the R statistical software environment using the complete linkage method as described in http://nlp.stanford.edu/IR-book/html/htmledition/single-link-and-complete-link-clustering-1.html [15].

## Results

**Figure 1:**
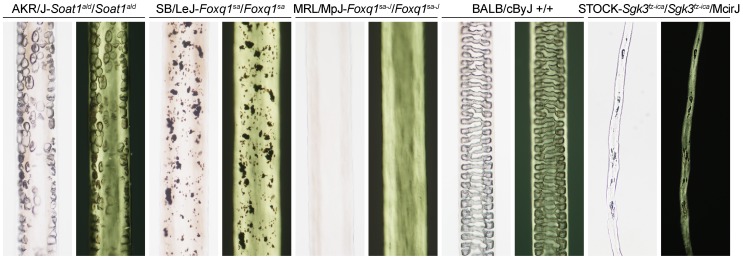
Hair visualized microscopically from 5 of the mouse strains analyzed. These are shown at the same magnification under brightfield (left) and polarized light (right). The appearances of strains not illustrated were similar to that of BALB/cByJ.

Hair shaft samples were analysed by shotgun proteomics from 11 mouse strains distributed among the 7 major groups in the mouse family tree [1]. In addition, 5 mutant stocks, one of which is an inbred strain, were compared. The mutant stocks had hair shafts with distinct structural (anatomic) differences from normal inbred mice with either pigmented or unpigmented (albino) hair (Figure 1).

### Analysis of Total Hair

**Figure 2:**
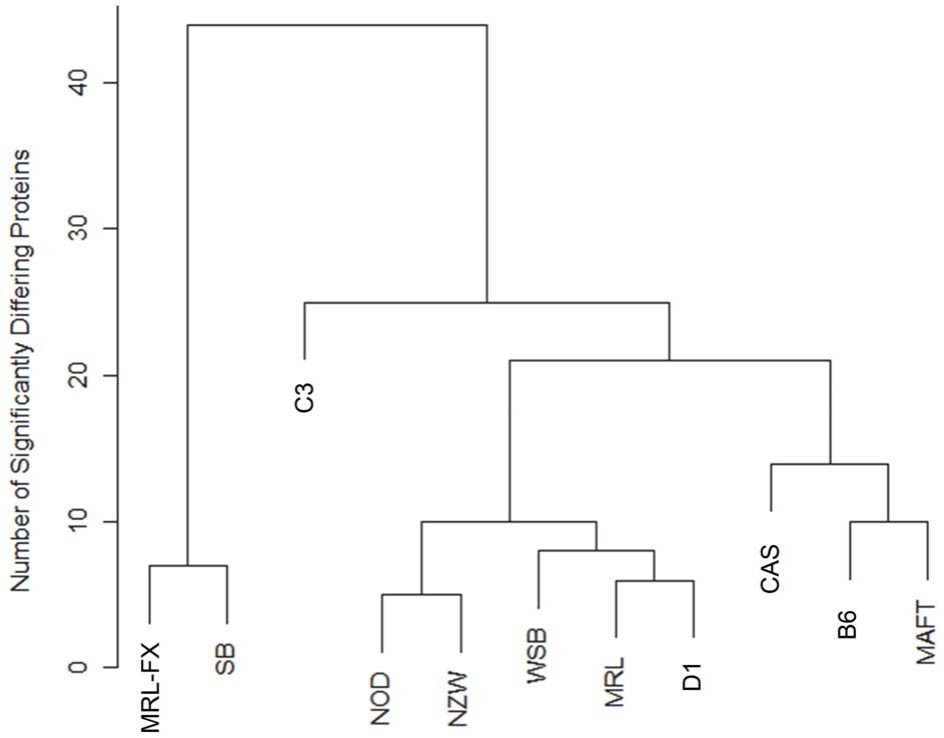
Hierarchical clustering of mouse strains based on relatedness according to protein differences in total hair.

Samples of total hair shafts were analysed from 8 inbred strains and 3 mutant stocks. Table S1 gives the spectral counts for the proteins identified in each sample. Statistical analysis identified 107 proteins with significant differences among the strains of which 94 permitted two way comparisons between strains (Table S2). As seen in the summary in Table 3, the strains and mutant stocks were readily distinguishable as indicated by a range of 5 to 44 pairwise differences. The data were used to generate a hierarchical clustering (Figure 2) that is not obviously related to previously established relationships among mouse family tree groups but does correspond to known hair defects within strains and mutant stocks. Particularly remarkable were the many differences distinguishing hair of the MRL-FX and SB mutants from the other strains, demonstrating the power to detect consequences of allelic mutations in the *Foxq1* gene yielding the satin phenotype which manifest as abnormalities in the hair shaft (Figure 1). There were few differences between hair from MRL and DBA strains and between NOD and NZW strains (groups 1, 6, 2 and 3, respectively).

**Table 3:** Numbers of proteins differing significantly among mouse strains.*

*Group*	*1*	*1*	*1*	*2*	*3*	*4*	*4*	*5*	*6*	*7*	*7*
Strain	C3	MRL	MRL-FX	NOD	NZW	B6	MAFT	SB	D1	CAS	WSB
C3	•	25	19	19	23	22	17	35	19	19	21
MRL		•	20	9	7	8	21	31	6	15	7
MRLFX□			•	18	16	31	27	7	12	42	23
NOD				•	5	10	14	28	8	17	10
NZW					•	14	17	20	5	21	7
B6						•	10	26	12	14	8
MAFT□							•	26	13	11	11
SB□								•	16	44	26
D1									•	17	8
CAS										•	14

* Shown in the matrix are the mouse strains, above each of which is the phylogenetic group to which it belongs [1].

□ Strains carrying single gene mutations affecting hair shaft anatomy.

**Figure 3:**
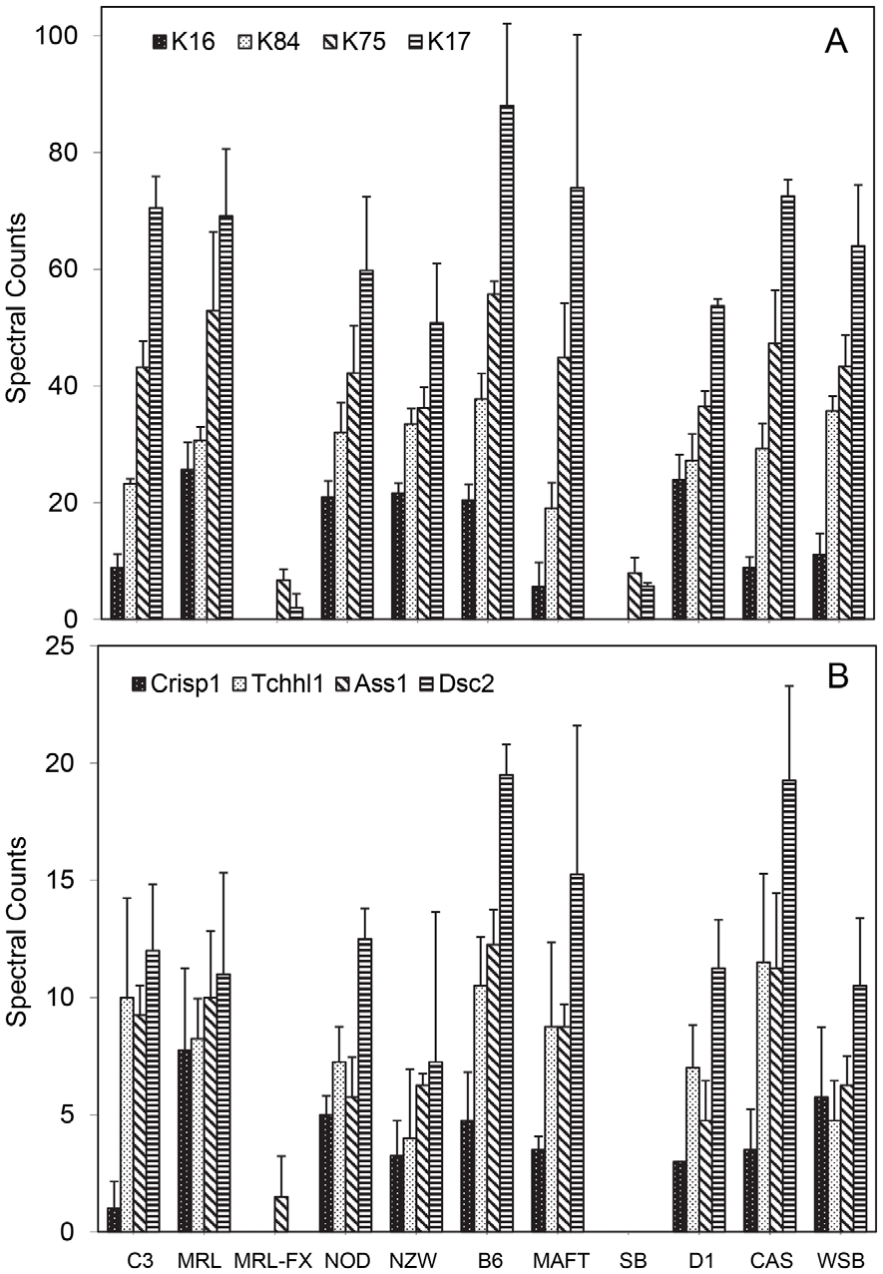
Distribution of KRT17, KRT33b, KRTAP6-1, and DSC2 among total hair shafts of mouse strains surveyed.

Figure S1 illustrates the top 50 constituent proteins by relative frequency in distinguishing the hair samples in two-way comparisons. Numerous prominent keratins were expressed at distinctive levels among the various strains. Specifically, KRT16, KRT17, KRT75, and KRT84 showed striking contrasts overall (Figure 3A). Especially conspicuous was the paucity of these 4 keratins in strains MRL-FX and SB, reflecting a common *Foxq1* gene defect. Figure 3B illustrates that proteins CRISP1, DSC2, ASS1, and TCHHL1 were also absent or nearly so in hair from the MRL-FX and SB strains. This contrast was evident in the spectral counts prior to adjustment for shared peptides (shown in Figure S2), where the values were generally within 10% of those after adjustment with an occasional exception such as for TCHHL1. At least 5 prominent keratins (KRT31, KRT33B, KRT34, KRT81, and KRT83) with distinctive levels among strains were not markedly lower in MRL-FX and SB strains. Similarly, the proteins encoded by DSG4, DUSP14, SFN, and TGM3 genes were moderately variable among strains but were not deficient in MRl-FX and SB mutants (Figure S3).

**Figure 4:**
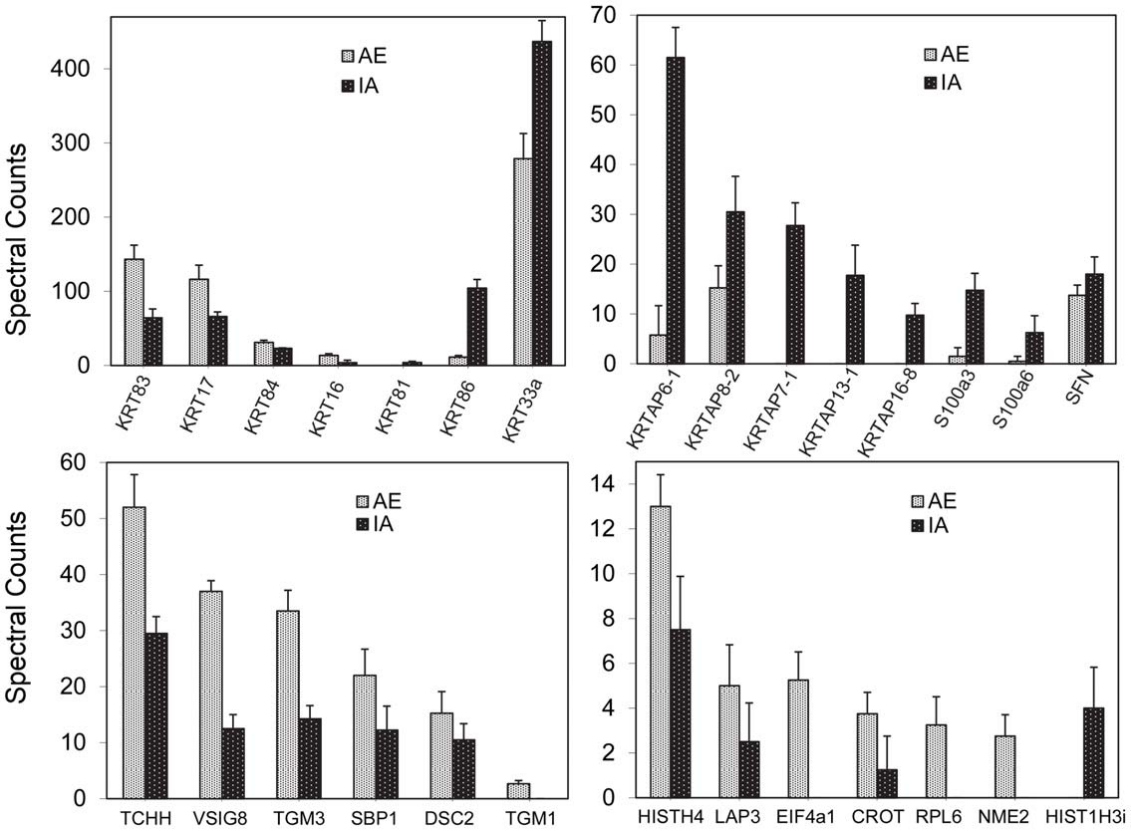
Comparison of yields of assigned spectra (not corrected for shared peptides) in C3 mouse hair. Cysteines were alkylated by iodoacetamide (IA) or 2-bromoethylamine (AE).

In the course of this work, the possibility of strengthening the identification of constituent proteins by alkylation of cysteines with 2-bromoethylamine was investigated. By providing extra tryptic cleavage sites in proteins with suboptimal levels of Arg and Lys, aminoethylation can increase the yield of peptides and thereby assist identification of protein components [16]. Moreover, chemical cleavage at cysteines using 2-nitro-5-thiocyanobenzoic acid has been found to increase the peptide coverage of KRTAPs in sheep wool (Koehn et al, 2009). In the present work with hair from the C3H/HeJ (C3) strain, as shown in Figure 4, the yield was increased in more proteins than it was decreased. However, the yield of KRTAP peptides was greatly decreased, probably due to their high cysteine content and the consequent generation of small peptides below the scan range of the mass spectral method when subjected to additional cleavage at basic residues. In view of the loss of KRTAP identifications, standard iodoacetamide alkylation of cysteines was preferred to aminoethylation. The possibility of increasing protein identification using both modifications in future work was noted.

### Analysis of Particulate and Solubilized Fractions

Pelage hair samples from 5 inbred strains and 5 mutant stocks, partially overlapping those above, were analyzed by shotgun proteomics after separating the solubilized fraction (largely keratins and keratin-associated proteins) and the particulate (insoluble) isopeptide cross-linked fraction. The identified proteins for each sample are given in Tables S3 and S4. Most of the highly prevalent proteins in the solubilized fraction were also found in the particulate fraction as previously described for human hair [17]. The proportions in each fraction varied; illustrating the extremes, little KRT19 was found in the particulate fraction, and little trichohyalin in the soluble fraction.

In an early stage of analysis, it became evident that global analysis of the individual soluble and particulate fractions was considerably less discriminating than that of the unfractionated hair shaft. Previous work indicated fractionation enhanced the detection of less prominent components in the cross-linked fraction, but this was overbalanced by the larger variance among samples. The observation that the strains can be readily distinguished without fractionation will considerably simplify future analysis. Although for this reason the spectral counts were not given a completely parallel workup (e.g., searched using the comparable IPI database instead of Uniprot and not adjusted for shared peptides), numerous strains were distinguishable in pairwise comparisons (Tables S5 and S6).

**Figure 5:**
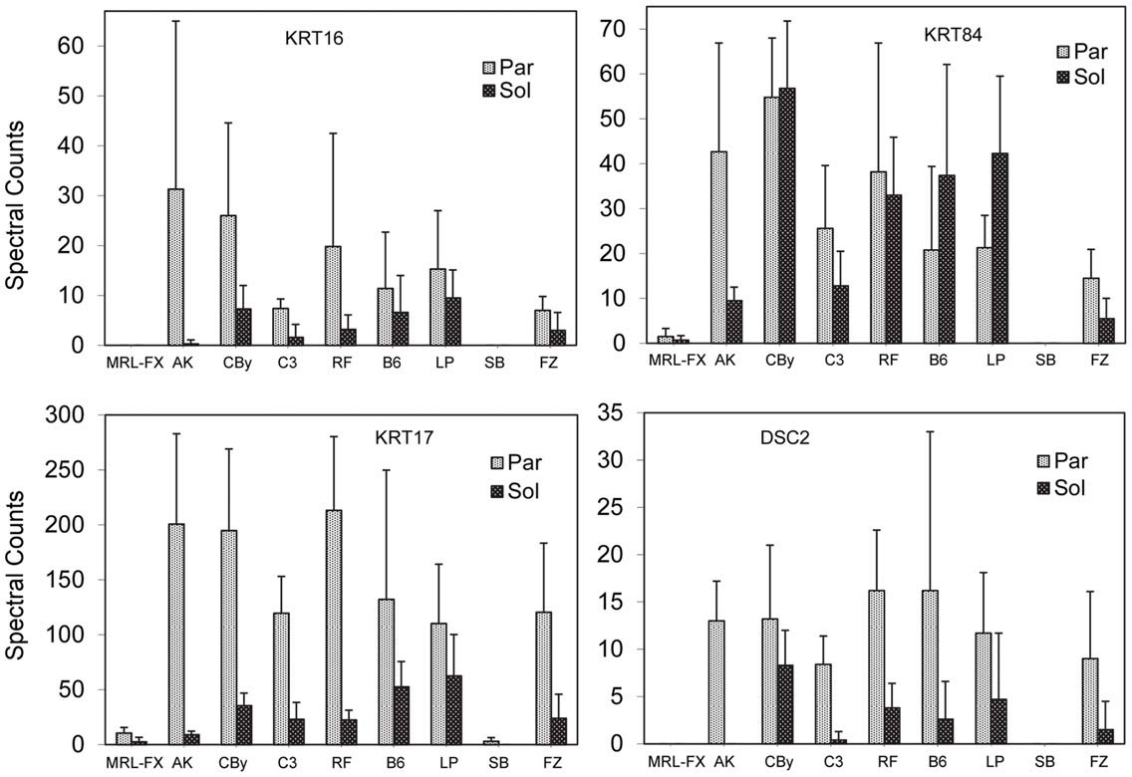
Profiles of KRT16, KRT17, KRT84, and DSC2 among hair shaft fractions of mouse strains surveyed.

The most discriminating proteins among both fractions were KRT84, KRT17, KRT75, and VSIG8 (Table 4). This analysis confirmed the paucity of KRT16, KRT17, KRT84, and DSC2 in the MRL-FX and SB mutants (Figure 5). As illustrated in the compilation of pairwise comparisons (Table S7), analysis of the particulate fraction was more discriminating than the soluble fraction. Hierarchical clustering on this basis (not shown), similar to that from total hair, grouped the MRL-FX and SB mutants far from the rest and the FX mutant nearly as distant from the inbred strains, including its wildtype control MRL/MpJ. All members of the AKR/J inbred strain are homozygous for the hair interior defect mutation (*Soat1^ald^*) [18]. Hair from these mice displayed few differences from its close relative, RF/J (according to Petkov et al, 2004) in pairwise comparisons, and the clustering placed it nearby. This may reflect the observation that while the larger pelage (guard) hairs often have a prominent defect, not all hairs are affected, especially not the predominant small zigzag hairs.

**Table 4:** Proteins discriminating among fractionated hair shafts of mouse strains surveyed.*

Protein	Part	Sol
KRT84	6	16
KRT17	10	6
KRT75	7	9
VSIG8	8	3
KRT82	5	1
TCHH1	6	0
SFN	1	5
KRT86	5	0
KRTAP6-1	4	0
KRTAP15-1	4	0
DUSP14	0	3
KRTAP13-1	3	0
KRTAP14	2	0
S100A3	1	0
FABP4	1	0
ASS1	1	0
KRT39	1	0

* The insoluble particulate (Part) and solubilized (Sol) fractions were analyzed separately and the results subjected to statistical testing.

## Discussion

Visualization of mouse hair under bright field and polarized light microscopy can reveal differences in shaft structure among inbred strains. As illustrated in Figure 1, contrasts can be dramatic as a result of mutations in specific genes. Two allelic mutations of the mouse *Foxq1* gene (*Foxq1^sa^* and *Foxq1^sa-J^*) [19] and one for the *Sgk3* gene (*Sgk3^fz-ica^*) [20] studied in this work had hair shaft medullas that do not form properly. Present work using these strains, pursuant to previous results showing a trichohyalin deficiency in the hair interior defect mutant phenotype in all AKR/J mice [4], provides a proof of concept that major structural abnormalities of the hair shaft are reflected in their proteomic profiles. In the case of hair with the *matted* mutation, of uncertain gene identity but which cosegregates with a filaggrin mutation [21], electron microscopic examination permits visualization of severe cuticle defects [22]. The protein profile differences between this strain and its closest relative, C57BL6J, were not dramatic, suggesting analysis of isolated cuticle cells could be more illuminating as to the basis for the observed defect. In such work, one must recall that mice have 4 hair shaft types on their body and many more on their ears, eyelids, etc. [23] that can be affected differently by alterations in gene expression [24], [25]. Not all of these may display microscopic abnormalities, as is the case with homozygotes for either the *Soat1^ald^* or *Sgk3^fz-ica^* mutation.

We demonstrate here differentiation of inbred mouse strains and normal, wild type, inbred strains from those same strains with single gene mutations that cause hair shaft defects. While some of the strains have known defects, such as AKR/J [18], homozygous for the hair interior defect mutant (*Soat1^ald^*), others appear to have normal hair. Among the latter, C3H/HeJ has normal appearing hair but is prone to development of alopecia areata, an autoimmune hair follicle disease characterized by defective hair shafts that break off [26]. While alopecia areata has a very late onset, usually over 6 months of age, C3H/HeJ mice are clearly separated from the other common “normal” inbred strains in Figure 2. Even in diseases arising from different allelic mutations in the same gene (and with each allelic mutation being on a different inbred strain background), the pattern of protein change was nearly identical, suggesting it is possible to develop hair proteomic profiles for specific disease diagnosis, perhaps subtyping these diseases, and possibly, in the case of C3H/HeJ and alopecia areata, predicting susceptibility to disease as a prognostic marker. As some of these mutations are fixed in these inbred strains, the profiling could serve as a diagnostic tool to screen strains for novel diseases as well as distinguishing the strains. By analogy, this is anticipated to work equally well for evaluating human hair.

An advantage of the shotgun approach is that numerous proteins are examined simultaneously in parallel. While only the most prevalent 50–100 proteins currently are identified at levels useful for the present analysis (likely to increase with increasing mass spectrometer sensitivity), these are sufficient to provide considerable discrimination. With few exceptions, most of the genes in this list are associated with protein expression in the skin and adnexa (Mouse Genome Informatics; http://www.informatics.jax.org/). Those with no known function may well be found to be expressed in these locations. For example, this shotgun approach has led to recognition of VSIG8, a previously undescribed protein, as a prominent component in hair shaft and nail plate [27]. Ongoing phenotyping of the many genetically engineered mice being created in the international knock out mouse project (KOMP2) [28]–[30] is anticipated to provide information in the near future on effects of specific genes, possibly including a variety of metabolic diseases that might affect hair quality. This would complement analysis of strains observed directly to have hair defects. Finding proteins to be missing in a given strain may offer a strong clue to observed alterations in hair structure. While phenotypic differences may be visible in photomicrographs of hair samples from some of the mice in this study (Fig. 1; http://www.pathbase.net/), more subtle differences may be observed in general only by transmission electron microscopy after detergent extraction [22] or by immunohistochemistry [27]. Absence of one or more proteins, even those ordinarily below the limit of detection, may be manifest in altered profiles of other proteins. For example, the striking effects of mutations in *Foxq1* likely reflect its function as a transcription factor altering many downstream proteins. Pursuit of such findings could include determining whether the lack of expression is tissue-specific or a result of a defect in the gene itself. Point mutations that do not affect protein stability may not be detectable using this approach unless the relevant peptides are obtained in substantial yield and happen to be sought specifically. However, a much more sensitive targeted approach (often called multiple reaction monitoring) could be possible if certain mutations were suspected. Indeed, a future diagnostic paradigm could employ a panel of proteotypic peptides from proteins known to be altered in specific diseases. To this end, a technical aspect needing attention is the considerable overlap in peptides among closely related proteins, such as the keratins and keratin associated proteins, leading to the present use of software that sorts the peptides better to minimize the overlap.

These results raise the possibility that cut hair, used for decades to test for heavy metals and drugs [31], [32] or structural diseases [5], [7], can now help diagnose complex diseases and possibly monitor efficacy of treatment for these diseases over time. As mice and humans have a high degree of homology between their genomes and share most of the same diseases, both species can be studied and compared [33]. Mice have the advantage that large numbers of inbred strains are available, many of which are now having their genomes completely sequenced and annotated, with well-defined mutations that directly or indirectly affect the hair. These strains provide monomorphic populations to test the efficiency of analytical methods before progressing to the more challenging highly pleomorphic human population.

## Supporting Information

Figure S1 **Ranking of proteins by their frequency in distinguishing among strains in two-way comparisons.**
(TIF)

Figure S2 **Distributions of keratins 16, 17, 75 and 84 (A) and CRISP1, TCHH1, ASS1, and DSC2 (B) among the strains.** Spectral counts were not adjusted for shared peptides. Comparison with Figure 2 reveals the adjustments were small except for TCHHl1.
(TIF)

Figure S3 **Profiles of keratins and other proteins not deficient in MRL-FX and SB mutant strains.**
(TIF)

Table S1 **Analyses of total hair shaft.** Shown are assigned spectra adjusted for shared peptides grouped in strains with the mean and standard deviation for each.
(XLSX)

Table S2 **Proteins in total hair shaft samples for which global p values for differences are <0.05.** P values are also given for two way comparisons, where those <0.05 are highlighted. For each comparison, the direction of the difference (up/down) is also given. The proteins are listed in order of most to fewest significant differences.
(XLSX)

Table S3 **Assigned spectra from particulate fraction of hair shaft samples.** Given are the identified proteins, their accession numbers and molecular weights, and number of assigned spectra for each hair sample, grouped in strains with the mean and standard deviation for each.
(XLSX)

Table S4 **Assigned spectra from soluble fraction of hair shaft samples.** Given are the identified proteins, their accession numbers and molecular weights, and number of assigned spectra for each hair sample, grouped in strains with the mean and standard deviation for each.
(XLSX)

Table S5 **Proteins in insoluble (particulate) fractions of hair shaft samples for which global p values for differences are <0.05.** P values are also given for two way comparisons, where those <0.05 are highlighted. For each comparison, the direction of the difference (up/down) is also given.
(XLSX)

Table S6 **Proteins in solubilized fractions of hair shaft samples for which global p values for differences are <0.05.** P values are also given for two way comparisons, where those <0.05 are highlighted. For each comparison, the direction of the difference (up/down) is also given.
(XLSX)

Table S7 **Proteins displaying significant differences in expression level in pairwise comparisons after separation of the hair into solubilized and particulate fractions.**
(XLSX)
