# Supplementary figures and images for: Differentiating Inbred Mouse Strains from Each Other and Those with Single Gene Mutations Using Hair Proteomics
Source: PLoS One. 2012 Dec 14;7(12):e51956. doi: 10.1371/journal.pone.0051956 (PMC3522583; doi:10.1371/journal.pone.0051956)

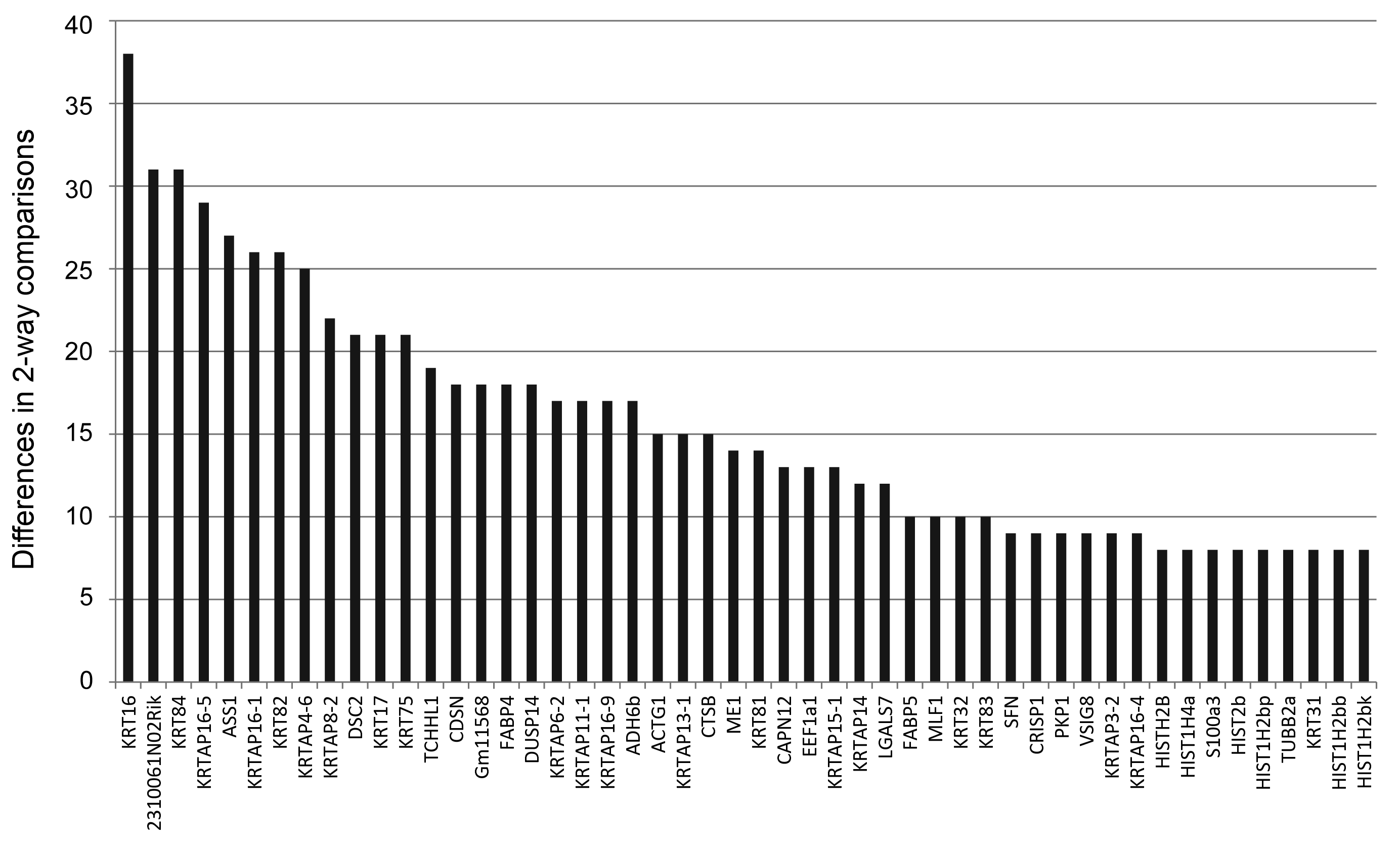

Supplement: Figure S1 — Ranking of proteins by their frequency in distinguishing among strains in two-way comparisons. (TIF) [file pone.0051956.s001.tif]

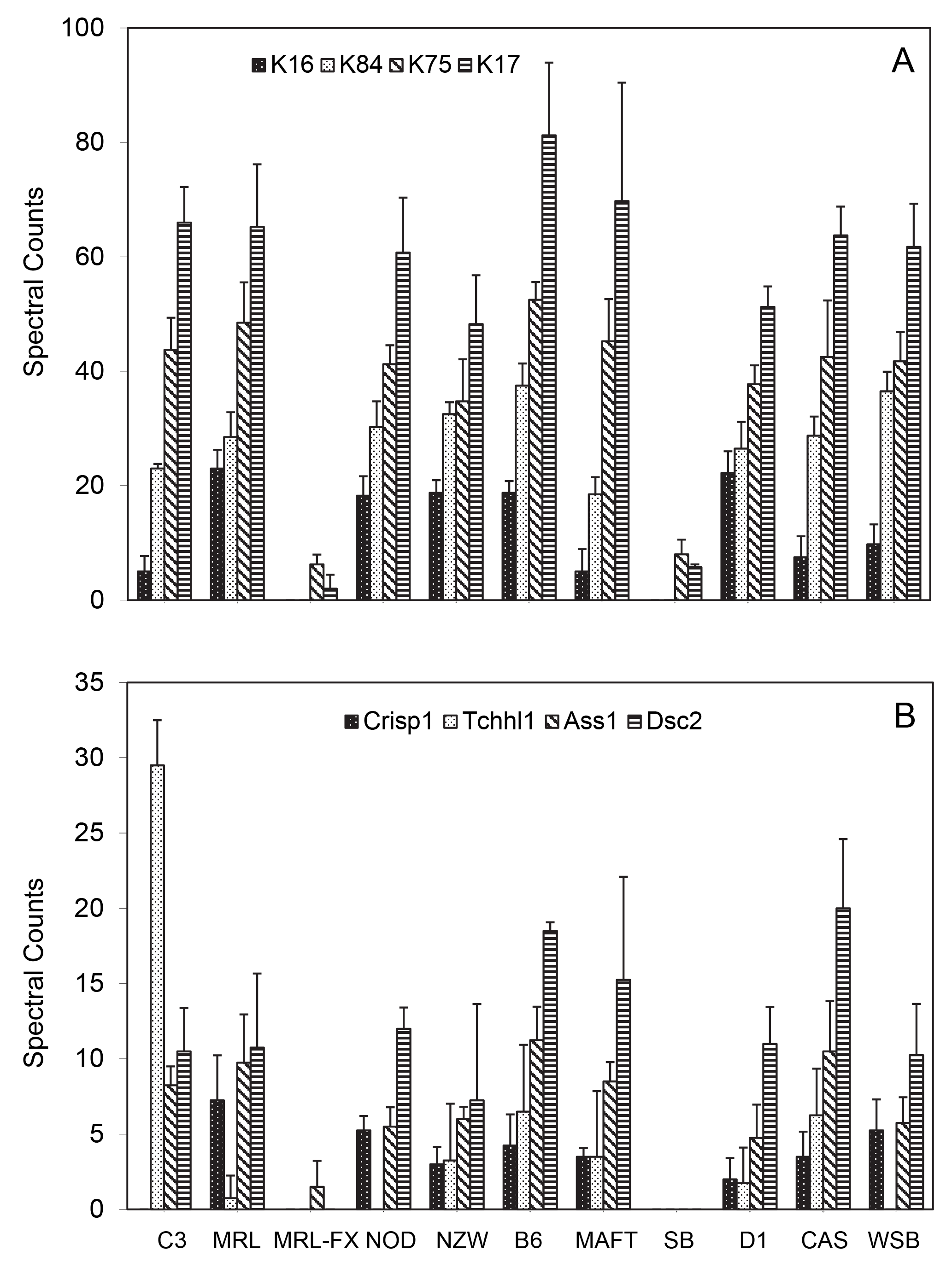

Supplement: Figure S2 — Distributions of keratins 16, 17, 75 and 84 (A) and CRISP1, TCHH1, ASS1, and DSC2 (B) among the strains. Spectral counts were not adjusted for shared peptides. Comparison with Figure 2 reveals the adjustments were small except for TCHHl1. (TIF) [file pone.0051956.s002.tif]

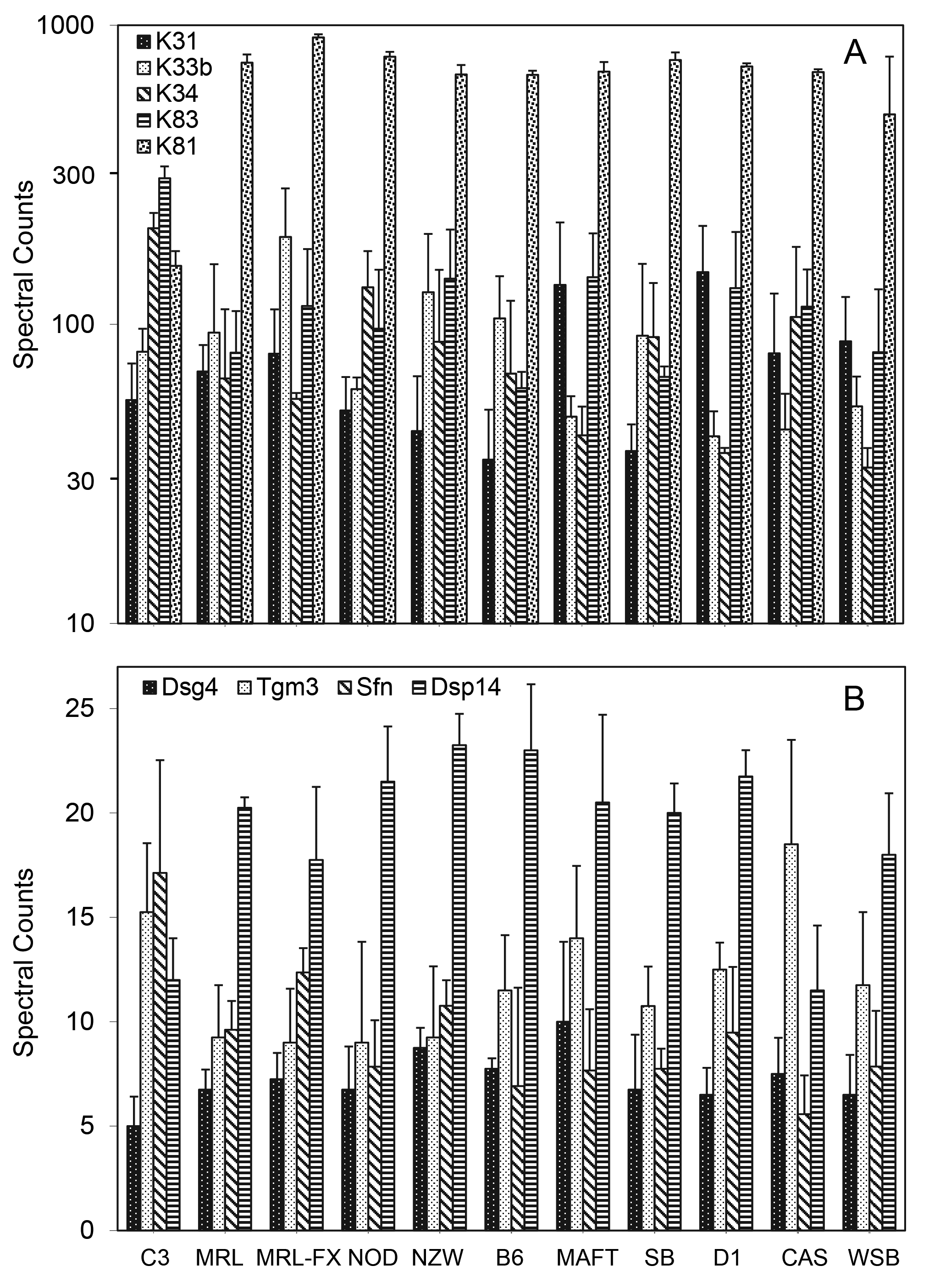

Supplement: Figure S3 — Profiles of keratins and other proteins not deficient in MRL-FX and SB mutant strains. (TIF) [file pone.0051956.s003.tif]
